# Analysing Credibility of UK Social Media Influencers’ Weight-Management Blogs: A Pilot Study

**DOI:** 10.3390/ijerph17239022

**Published:** 2020-12-03

**Authors:** Christina Sabbagh, Emma Boyland, Catherine Hankey, Alison Parrett

**Affiliations:** 1 Human Nutrition, School of Medicine, Dentistry and Nursing, College of Medical, Veterinary & Life Sciences University of Glasgow, New Lister Building, Glasgow Royal Infirmary, Glasgow G31 2ER, UK; Catherine.Hankey@glasgow.ac.uk (C.H.); Alison.Parrett@glasgow.ac.uk (A.P.); 2 Department of Psychology, University of Liverpool, Bedford Street South, Liverpool L69 7ZA, UK; e.boyland@liverpool.ac.uk

**Keywords:** social media influencers, blogs, weight management, nutrition, evidence-based

## Abstract

Social media influencers (SMI) are individuals with large follower engagement, who can shape the thoughts and dietary behaviours of their audience. Concerns exist surrounding the spread of dietary misinformation by SMI, which may impact negatively on public health, yet no standards currently exist to assess the credibility of their information. This study aimed to evaluate the credibility of key SMI weight management (WM) blogs (*n* = 9), piloting a pre-prepared credibility checklist. SMI were included if they had a blue-tick verification on ≥2 social media (SM) and an active WM blog. A sample of blog posts were systematically evaluated against thirteen credibility indicators under four themes: ‘transparency’, ‘use of other resources’, ‘trustworthiness and adherence to nutritional criteria’ and ‘bias’. Indicators were yes/no questions to determine an overall credibility percentage for each SMI. The ten most recent meal recipes from each blog were evaluated against Public Health England’s (PHE) calorie targets and the UK ‘traffic light’ food labelling scheme to assess nutritional quality. Percentages ranged from 23–85%, the highest gained by a Registered Nutritionist. SMI blogs may not be credible as WM resources. Given the popularity and impact of SM in the context of overweight, obesity and WM, this study may inform the methodological approach for future research.

## 1. Introduction

Obesity is a global health concern, affecting an estimated 650 million adults worldwide [1]. Foods high in fat, sugar and/or salt (HFSS) are readily available, accessible and accepted, contributing to obesogenic environments, a key driver of population weight gain [2]. Implementing governmental policy and legislation may improve obesity-related population health [3]. Links exist between digital marketing of HFSS products, their intake, and overconsumption in children [4], with online HFSS marketing becoming a target for policy intervention. However, SM remains under-legislated, with loopholes existing in current marketing regulations [5]. SM is defined as ‘websites and computer programs that allow people to communicate and share information on the internet using a computer or mobile phone [6]’, and in the UK there are currently around 45 million active SM users [7]. SMI have thus emerged, so-called due to the effect their opinions have on persuading their audience [8], often to purchase products or join movements [9] or follow their advice, connecting through shared ideals, and shaping attitudes and behaviors [10]. Recently, brands have favoured SMI marketing, spending an estimated USD 8 billion globally in 2019, projected to increase to USD 15 billion by 2022 as popularity grows [11].

SMI connect with their ‘followers’ through both SM and blogs: regularly-updated websites, often with widely shared opinion-based articles on specific topics. In the UK, ‘Nutritionist’ is not a protected title, meaning that there are no legal restrictions as to whom may use the title, or any safeguards to ensure that those who do use it have the required training and qualifications. This makes it difficult for the public to differentiate between a qualified and non-qualified nutritionist. Influencers sharing nutrition and WM information need not be qualified Nutritionists or Dietitians, raising concerns about the credibility of the available information. The consequences of the public following opinion- versus evidence-based advice, coupled with the power of SMI, could be harmful, with SMI already implicated in cases of orthorexia nervosa [12], an obsession with eating a healthy, or supposedly healthy diet [13]. Evidence-based messaging from governments and public health organisations may also be lost, leading to confusion from contradictory advice, rendering it difficult for consumers to make informed, healthy choices.

Celebrity endorsement of food choice has previously been linked to overconsumption in children [14]. Vlogger endorsement of snacks affected children’s ad libitum snack intake, with those randomised to view still images of SMI with unhealthy snacks consuming 26% more calories than those randomised to view influencers with non-food products (*p* = 0.001), and 15% more than those viewing influencers with healthy food (*p* = 0.05), with more calories consumed from unhealthy snacks [4]. Interestingly, viewing the same SMI with healthy foods did not lead to children consuming more healthy snacks. Although adult susceptibility was not determined by these studies, the psychological power of ‘celebrity/influencer’ is highlighted, demonstrating the need for the accuracy and consistency of nutritional information shared.

One means of assessing content posted online, the technique of SM content analysis, has been used in research across several areas of public health, including for the assessment of general communication of breastfeeding promotion online [15]. Content analysis using Twitter has also provided researchers with insight into general public discourse surrounding prominent policy areas, including minimum unit pricing for alcohol [16] and standardised packaging of tobacco [17], which can help to predict the general public’s attitude to public health policy changes. Content analysis has also been applied to health blogs, with a recent study following five blogs over a 3-month period to determine the common characteristics of healthy eating blogs [18]. The study identified that communicating the main purpose of each blog post immediately (e.g., in the title of the post) was the main factor in determining health blog success, reflecting advertising research that suggests prominent headings that denote a perceived benefit (such as those found on healthy eating blogs) are the most engaging [19]. Whilst this research is limited by its focus on Australian blogs, similar techniques may be used by bloggers globally.

Similarly, researchers have analysed healthy eating blogs to determine the difference in the nutritional quality of recipes and credibility of blogs by qualified and non-qualified individuals, with varying results. A recent analysis of 75 nutrition-related blogs noted no statistically significant differences in the provision of references, disclaimers or promotion of services between qualified and unqualified nutrition bloggers, although evidence-based referencing was used more frequently in the qualified group [20]. The nutritional content of recipes, however, has been found to be more adequate for certain nutrients in recipes posted by qualified professionals, compared to non-qualified bloggers in the same field [21], as has overall credibility [22].

Whilst similar areas have been explored in relation to SMI in recent years [23,24,25,26], it is novel to apply content analysis to the topic of the current study. Little to no evidence exists surrounding the use of a checklist of quality indicators that can be used to assess the credibility of WM blogs authored by SMI, combining both credibility elements of information-based posts and nutritional analysis of recipes. Due to there being no current UK regulations as to who can post WM advice and information online, it is imperative that a means of assessing the credibility of blogs is developed with an aim to halt the spread of related misinformation online, which, given the prominence of SMI and users, has the ability to interfere with official public health messages and guidance. This study aimed to assess the credibility of WM blogs as appropriate resources for WM, by use of a pre-prepared checklist and systematic coding, offering a preliminary assessment of the patterning of WM advice provided by SMI online, whilst piloting a credibility checklist for use in this context. Several indicators on the checklist required the evaluation of individual blog posts (for example, when assessing the use of evidence-based references) for which ‘information-based’ posts were identified and selected.

To further understand the appropriateness of the blog content for WM purposes, the calorie content and nutritional quality of WM influencers’ meals were evaluated against PHE’s ‘One You’ calorie targets [27], and Food Standards Agency’s (FSA) traffic light food labelling scheme [28]. This evaluation first required the identification of meal recipes (10 per influencer, categorised into breakfast, lunch and evening meals), which were then analysed using dietary analysis software, as described in methods.

## 2. Materials and Methods

### 2.1. Criteria for Standardised Credibility Checklist

Blogs and individual posts of SMI were coded systematically, by a single coder, against a pre-prepared checklist of credibility indicators. To define the checklist, conclusions of a systematic review which shortlisted key ‘quality’ indicators of health educational blogs and podcasts, guided the elements chosen [29]. The indicators identified under the theme of ‘credibility’ were consensus checked to SMI and considered appropriate for this work. This final checklist was adapted to fit the current study. Thirteen indicators and four themes were identified: ‘transparency’, ‘use of other resources’, ‘trustworthiness and adherence to nutritional criteria’ and ‘bias’ (Table 1). Yes or no questions (Supplementary Table S1) were used, but a ’zero’ (0) response was recorded when the content of the blog did not contain information pertinent to the question. For influencers who recorded one or more ‘0’ responses, the corresponding indicators were subtracted from the total number of indicators and scores calculated. For example, if an influencer recorded three ‘0’, instead of calculating the score out of thirteen, it was calculated out of ten, thus avoiding negative marking for not including a particular element.

Overall credibility percentages were calculated (e.g., a ‘yes’ for 6 of the 13 indicators would have been awarded 46%), for comparison between influencers.

### 2.2. Identification of Influencers

Influencers were identified using the SMI marketing website ‘influence.co’ [30], filtered by ‘United Kingdom’ and ‘nutrition’. Influence.co is a search-engine-style platform designed to allow SMI to list their services to marketers, and for marketers to easily identify SMI in their field. Over 100,000 SMI are listed on the platform, with similar platforms used in previous research [21]. This was followed by Instagram and Google Incognito searches (removing the influence of previous searches) using combinations of the terms ‘nutrition’, ‘diet’, ‘physical activity’, ‘weight management’, ‘obesity’, ‘blog’ and ‘influencer’.

The inclusion criteria required SMI to be UK-based with a currently active WM blog with at least one new post per month, reflecting a similar previous study [20]. Whilst traditional blogs are not a category of SM, SMI often use blogs as a forum for sharing more detailed information, and therefore were selected for evaluation against the credibility checklist.

**Figure 1:**
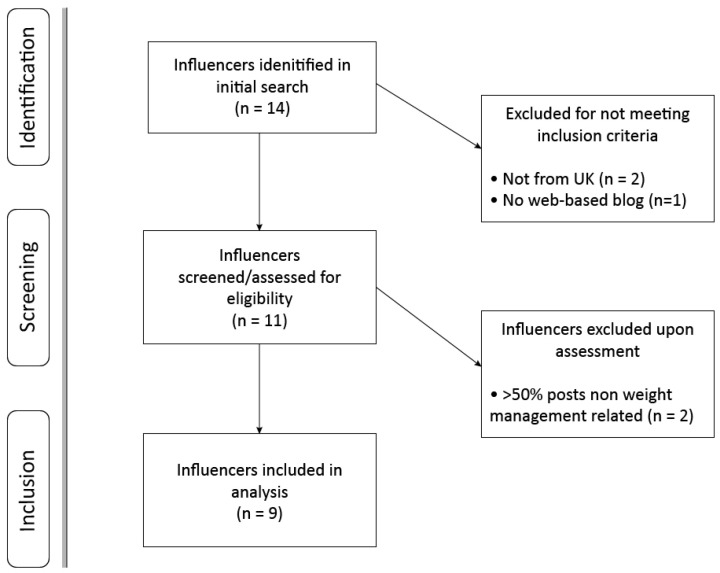
Flow diagram of influencer selection process.

A ‘blue tick’ verification on at least two SMs that offer it (Twitter, Facebook and/or Instagram) was required for an SMI to be included. While anyone can apply for such an account, blue ticks operate to verify the authenticity of ‘public interest’ accounts, and are commonly used to demonstrate industry influence of an individual, giving them a stronger online presence [31]. Blogs were only included if they focussed on WM, evaluated as >50% of posts relating to diet, nutrition or physical activity: core components recommended for effective WM interventions [32]. The 50% cut-off reflected a previous online credibility study [20]. Follower count alone does not denote influencer status [33], and ‘micro-influencers’ with between 1000 and 100,000 followers are considered important as they tend to focus on a niche area [34,35]. To capture these topic-specific SMI, follower counts across Twitter, Facebook and Instagram were used, with the lower cut-off being 80,000 on one SM. Nine UK influencers met all inclusion criteria and were included (Figure 1). Influencers’ names have been anonymised for legal reasons and are instead referred to by codes from SMI 1 to 9.

### 2.3. Selection of Meal Recipes

The most recent entries for three breakfasts and lunches and four evening meals (ten meal recipes) were selected from each SMI to ensure current output. If meals were listed under a combined ‘lunch and dinner’ category, they were randomly split into each meal category for analysis. As each SMI provided an intended number of servings, nutrition data were generated per serving. Recipes were viewed as meals, as the SMI either included recipes for each component of the meal, e.g., for curry, rice and salad, in the post, or, if the SMI specified the meal was served alongside rice, this was added as a standard serving [36]. This allowed for comparison to PHE meal calorie targets. Snacks were not considered, as not all SMI provided snack recipes, and limited information was provided by PHE to support additional analysis.

### 2.4. Selection of Information-Based Posts

The twenty most recent blog posts, reflecting the current activity and advice offered, from each SMI, were analysed from information-based sections of blogs. Where more than one information-based section was available, selection was split across relevant sections, e.g., the first ten posts each from ‘exercise tips’ and ‘nutrition advice’. SMI were required to cite evidence-based references on ≥70% of the sample to obtain a ‘yes’, consistent with the nutrition indicators. A reference was deemed to be evidence-based if it could be followed to a publication in a peer-reviewed journal or trusted public health source, e.g., PHE, and contained up-to-date information. References linking to opinion-based sources, such as other blogs, were not considered evidence-based. References leading to broken links or cited within the text but not present within the reference list were rejected.

### 2.5. Blog Characteristics and Influencer Demographics

‘About me’, ‘FAQ’ and ‘Disclaimer’ sections on blogs allowed the identification of professional affiliations, qualifications, age and sex of SMI. Blogs were analysed to determine if SMI advocated specific diets, food philosophies, personal stories or displayed nutritional information alongside recipes.

Medical doctors were considered qualified to provide WM advice, as nutrition is taught within the medical undergraduate curriculum in the UK, though course content varies. They are often the first point of contact for those seeking WM advice, and are perceived as being the most educated and credible profession [37]. Moreover, UK clinical guidelines [32,38] advise doctors to monitor weight and provide WM advice. Currently, ‘nutritionist’ is not a protected title in the UK; however, the Association for Nutrition (AfN) [39] comprises a register of degree-qualified nutritional professionals, who must meet rigorous evidence-based nutrition competencies. Therefore, alongside Registered Dietitians (protected title), AfN-Registered Nutritionists were also considered qualified. However, it cannot be assumed that they have specific WM expertise.

### 2.6. General Data Protection Regulation

Privacy statements and disclaimers were also scrutinised prior to, and one month after, implementation of the EU General Data Protection Regulation (GDPR; 25 May 2018). GDPR guidance protects EU citizens from data breaches; consumers must give their clear consent to the storage, processing and use of their data [40]. Clear and accessible privacy policies on blogs ensure that readers are fully informed concerning their data.

### 2.7. Adherence to Nutrition Criteria

Meals were analysed using Nutritics dietary analysis software [41] by imputing raw ingredients (g or ml). Nutritics generates a list of ingredient options from international databases. If available, ingredients were selected from the UK database [42], otherwise the first option generated was selected. Meals were analysed for energy, macronutrient content carbohydrate, protein, fat, saturated fat, fibre, sugar and salt content (g and mg). Percentage macronutrient composition was calculated for each meal. UK dietary guidelines recommend carbohydrate, total fat, and saturated fat intakes of 50%, <35% and ≤11% of daily food energy (TE) respectively [43]. The reference nutrient intake for protein is 0.75 g/kg bodyweight per day in adults, making up the remainder of daily food energy intake. The data collected were analysed against these guidelines.

Nutritional information was compared against the PHE ‘One You’ campaign kcal targets of: 400 kcal breakfast, 600 kcal lunch, 600 kcal evening meal (the remainder assigned for snacks) [27]. For the two indicators relating to nutritional criteria of the ten meals from each SMI, a ‘pass’ cut-off had to be established, as there was no binary answer. Adherence cut-offs have not been explored in the previous literature in this context; therefore, these were determined by the research team, with the following justifications: a ‘yes’ was given if ≥70% of meals fell within the accepted boundary (+10% kcal), ensuring adequate preservation of snack calories. If meals reached the upper limit, snack allowance would be 240 kcal/day (for women); higher cut-offs leave the potential for over-consumption.

Meals were analysed to assess the number of red, amber and green lights on the UK traffic light labelling system [28]. Whilst not mandatory in the UK, most food and drink packaging contains front-of-pack traffic light labels to help consumers make healthier choices. These labels use green, amber and red colour-coding to, respectively, represent low, medium and high amounts of fat, saturated fat, sugar and salt in a product, alongside energy content.

Consistent front-of-pack labelling aims to educate consumers to make healthier choices, improve diet and control energy intake. This indicator was marked as ‘yes’ if ≥70% of traffic lights were green or amber across the ten meal recipes, consistent with the previous adherence cut-off.

All blog posts and meal recipes included in the study were published in 2018 and analysed between 9 May and 9 June 2018, during the time in which the study was conducted.

## 3. Results

### 3.1. Influencer Demographics

Two SMI were male and seven female, all aged 27–43 years. This reflects the most common age groups noted in previous studies of science bloggers [44], health bloggers [45] and a recent survey of UK influencers [46]. That the current study identified more than three times the number of female SMI than males is likely reflective of the current influencer demographic of the UK, with a 2020 survey of 482 influencers reporting 79% female respondents, with 20% male (1% reported ‘other’) [46]. Two self-identified as personal trainers, one as a medical doctor, two as chefs, one registered associate nutritionist with AfN, and one nutritional therapist. Two influencers did not provide this information, so occupations were unknown. Each influencer had at least seven methods of communication, including Facebook, Twitter, Instagram, Snapchat, Pinterest, YouTube, Google+, the blog itself and email newsletters.

### 3.2. Blog Characteristics

**Figure 2:**
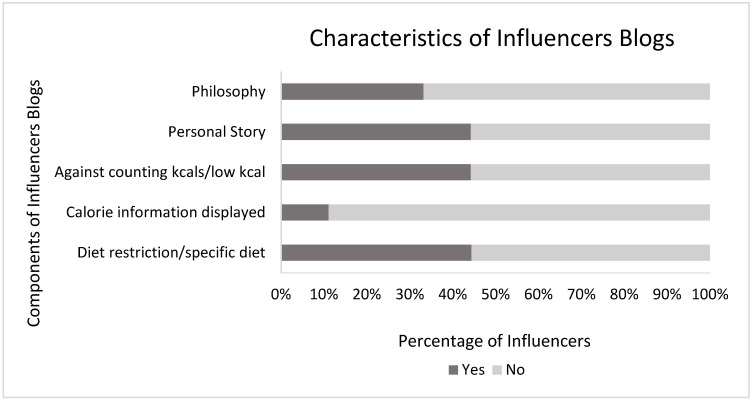
Components of influencers’ blogs (%). Dark grey represents the percentage of influencers who consider each component. Light grey represents influencers who do not consider each component.

Only one SMI provided calorie information, whilst four stated that they did not believe in counting calories, or low-calorie diets (Figure 2). Four female SMI advocated specific diets (Table 2), with rationales surrounding nourishment and healing. Similarly, four female influencers included personal stories on their blogs; three described their own ‘philosophy’ in relation to overall diet.

### 3.3. Credibility Assessment

In total, 180 blog posts were analysed, alongside 90 meal recipes.

#### 3.3.1. Theme 1: Transparency

Five influencers did not provide a disclaimer, and four did not include a privacy policy. SMI-6 had a link titled “recipe substitutions and disclaimer” in their blog footer, which did not lead to a disclaimer. Every post author was clear, and five SMI included a personal statement detailing their experience or knowledge in the field.

#### 3.3.2. Theme 2: Use of Other Resources

Five influencers did not cite references or evidence-based references when providing information in blog posts. Two (SMI-4 and 9) did not provide any relevant information-based posts, and so were marked ‘0’, meaning only two SMI cited evidence-based references on posts. SMI-5 provided no references on any posts analysed.

#### 3.3.3. Theme 3: Trustworthiness and Adherence to Nutritional Criteria

Only two influencers were considered qualified to provide WM advice: SMI-6 (Medical Doctor) and SMI-7 (Registered Nutritionist; AfN).

##### PHE Calorie Targets

Table 3 shows calories in meals, as mean and range of, and as a percentage of the PHE target. Breakfast had the lowest single meal pass rate, with over half (14/27) above the +10%kcal limit. SMI-5 provided the highest calorie breakfast, ‘Healthy English Breakfast’ at 1062 kcal: more than two and a half times the PHE target. Breakfasts within limits included a Bombay omelette, chocolate protein oats and green shakshuka.

Lunches ranged from 214 to 1024 kcal and evening meals from 234 kcal (SMI-6) to 1592 kcal (SMI-5). Lunches within limits included chicken fajitas, harissa prawn and feta salad and evening meals within limits included salmon spaghetti with greens, vegan mac and cheese, and tomato, red wine and chorizo risotto. SMI-5 consistently had the highest kcal count across all meal categories. SMI-7 had the highest number of meals within the limit. Of the four influencers who failed this indicator, three were those who stated that they did not believe in calorie-counting or low-calorie diets.

##### Traffic Light Labelling

**Figure 3:**
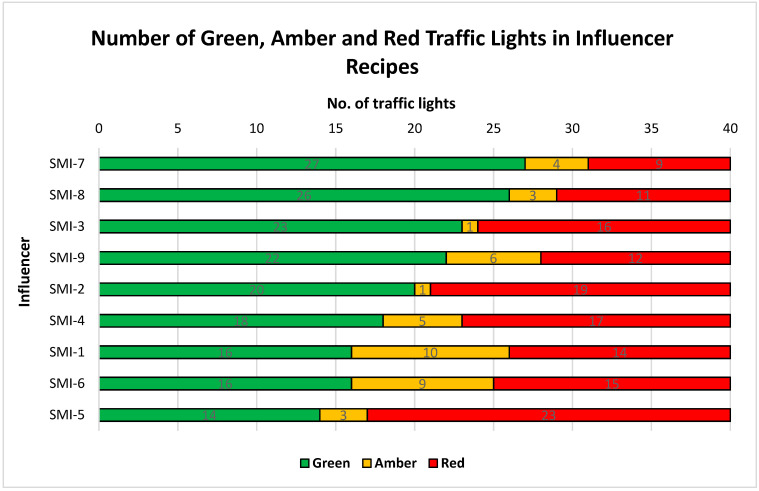
Number of green, amber and red traffic lights in influencer recipes. Only three influencers passed the traffic light criteria: SMI-7, SMI-8 and SMI-9, with 77.5%, 72.5% and 70%, respectively.

SMI-7 had the highest number of green lights (27/40); SMI-5 had the lowest (14/40). Similarly, SMI-7 had the lowest number of red lights (9/40), and SMI-5 the highest (23/40) (Figure 3). For eight influencers, red lights were mostly for fat/saturated fat. Green lights were mostly for sugar. Of the 90 meals analysed, only eight were awarded four green lights. SMI-5 and SMI-9 had a meal that contained four red lights.

Table 4 shows the percentage of nutritional criteria adherence by each SMI.

##### Macronutrient Analysis

Previous studies have reported a low-carbohydrate diet to be anywhere between 26% total daily energy from food (TE) [47] and <45%TE [48]. Using <45%TE cut-off, seven SMI appeared to advocate a low-carbohydrate diet. Eight SMI provided a high-fat diet (>35%TE). Protein lay within recommended levels (15–30%TE, depending on weight) for six SMI and above recommended levels for one SMI, whilst two fell short of recommendations, SMI-3 and SMI-9, each advocating vegan or vegetarian diets (Table 5).

#### 3.3.4. Theme 4: Bias

Six SMI did not distinguish between fact and opinion, providing no, or inadequate, references. Two did not disclose advertising, as identified through affiliate links. SMI-5 did not record a ‘yes/pass’ for any bias indicators.

### 3.4. Credibility Checklist Overall Pass Rate

**Figure 4:**
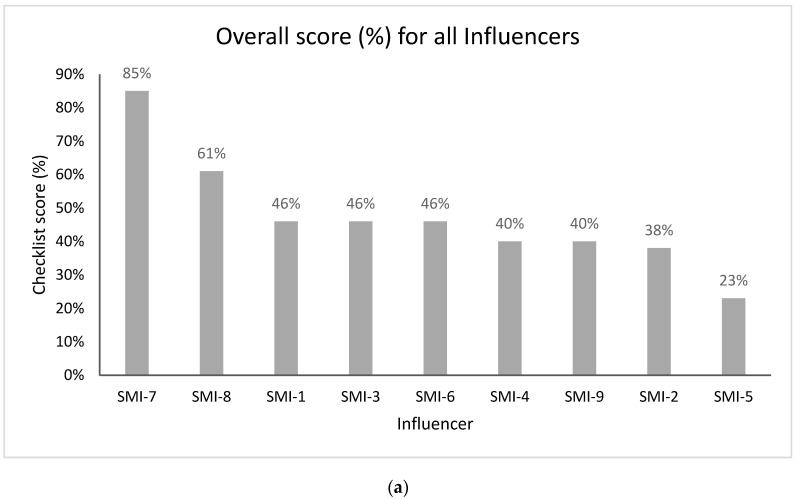

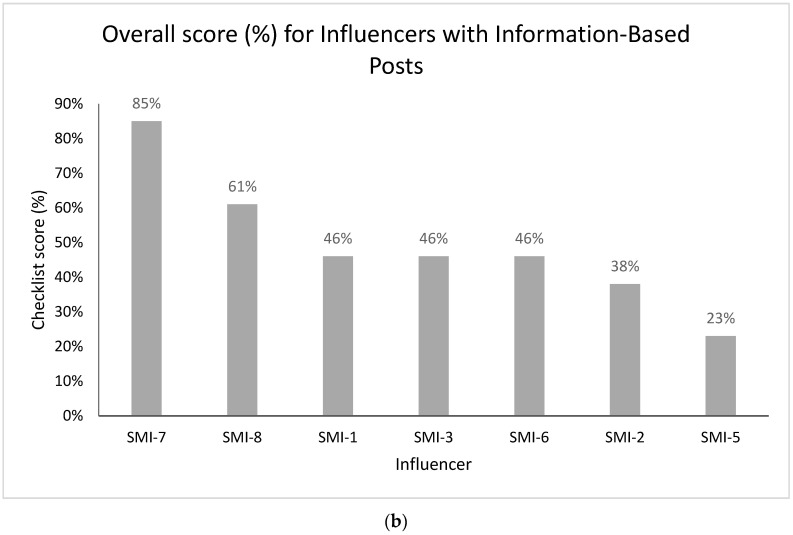
(**a**) Overall credibility score for all influencers (%) (*n* = 9). (**b**) Overall credibility score for influencers who provided information-based posts (%) (*n* = 7).

The registered nutritionist, SMI-7 scored the highest at 85%, followed by SMI-8 at 61% (Figure 4a). The final analysis was re-run to include only those who provided information-based posts (*n* = 7) (Figure 4b). Supplementary Table S1 provides a full breakdown of themes and quality indicators, alongside SMI scores.

## 4. Discussion

This study systematically assessed the credibility of UK SMI online WM information against evidence-based advice, piloting a pre-prepared credibility checklist. The aim was to offer an initial assessment of the patterning of this information online. The results suggest that SMI WM blogs are often not credible. Only two SMI were deemed qualified to provide advice; however, it is unclear whether any had formal WM training. The registered nutritionist was the highest scorer (85%), demonstrating that suitable qualifications might aid credibility.

The use of the internet to source nutrition information has risen in recent years, with an Australian study indicating that 40% of adult respondents used the internet as their main source of nutrition information [49]. Whilst not representative of UK habits, the UK government should consider collecting similar data in the National Diet and Nutrition Survey, to inform policy surrounding the provision of nutritional information online. Additionally, individuals following a gluten-free diet, as recommended by four influencers in the current study, have previously reported that they were recommended to follow the diet by a TV personality, blogger, vlogger or celebrity, significantly more so than individuals not following this diet [50]. Whilst this may suggest that in certain circumstances, individuals with restricted diets may turn to less traditional or credible information sources, the same study highlighted that the most common source of information cited by individuals following a gluten-free diet was family and friends, followed by healthcare professionals [50].

Anyone can share nutrition or WM information online, and therefore this may not be credible or accurate. The British Dietetic Association found that 58% of 2000 adults surveyed would trust a personal trainer or fitness instructor to give them nutrition advice, increasing to 75% for the 18–24 age group [51]. Worryingly, 41% of the same age group said that they would trust a ‘healthy eating blogger’, regardless of qualification.

Several extreme caloric values were recorded, e.g., SMI-5’s ‘Healthy English Breakfast’ contained 1062 kcal, more than half of a non-dieting female’s recommended daily caloric intake. The perceived ‘healthiness’ perhaps comes from no ‘processed’ foods being used. This does not automatically translate into a nutritionally healthy meal, and could hamper public WM efforts. Disclaimers present in blogs did not mention whether they were designed for weight loss, despite blogs offering weight loss advice and recipes, and use of words like “healthy” may confuse consumers’ ability to recognise reliable advice online.

NICE WM guidelines [52] recommend a caloric deficit of 500–600 kcal/day for weight loss in overweight and obesity, but more practical information is needed on how to implement the recommended deficit. Calorie-labelling has been previously associated with lower weight gain [53]; however, findings are not transferable to a ‘free living environment’. Only one influencer provided nutritional information. Online display of calorie and nutritional information in WM blogs may be suboptimal, not allowing for informed choice and tracking of consumption.

Though the concept of calories as a part of total daily energy intake may be too abstract for people to grasp [54], traffic light labelling is better understood, with products with a higher number of green lights six times more likely to be identified as ‘healthy’ [55]. Notably, traffic light labelling is not currently mandatory across the EU [56], leading to inconsistency in application, impacting consumers’ understanding and ability to use these labels to make informed healthier choices consistently. Whilst it is not currently mandatory in the UK, traffic light labelling is the preferred choice, and is implemented voluntarily by the industry. Following Britain’s exit from the EU, the UK Government held a public consultation on front-of-pack nutrition labelling [57], with policy and regulations therefore subject to change.

Additionally, portion sizes can substantially affect calorie consumption [58], although it is unclear whether blog readers follow given servings, as portion sizes are not standardised and difficult to assess [59]. Pictures on blogs can display larger servings and extra dishes, with the potential to mislead, again leading to overconsumption [60]. Offline, pictorial portion sizes have been found to be almost 65% larger than recommendations made on the same box [61,62], with recent findings demonstrating that front-of-pack portion size depictions influence children’s food intake [63]. Limited by the single product category in these studies, future research should aim to determine whether this occurs for other products and whether similar effects occur online.

### 4.1. Traffic Light Labelling and Macronutrient Composition

The most red lights were given for fat or saturated fat. Blog recipes have previously been found to exceed US recommendations for saturated fat and sodium, with vegetarian recipes, in comparison to meat, significantly lower in both [64]. NICE recommend a low-fat diet for weight loss, largely justified due to the high energy-density of fat (9 kcal/g) [52]. Additionally, some of the best evidence for weight-loss maintenance comes from those following low-fat diets, alongside regular self-weighing and physical activity [65]; however, this is based on self-reported data. A high-fat diet appeared to be advocated by eight SMI, as reflected by the nutritional analysis.

Protein is the most satiating of macronutrients: high-protein diets are therefore popular strategies for weight loss or maintenance [66]. The two SMI with meals below protein recommendations were both nutritionally unqualified and advocates of plant-based diets. This aligns with the findings of a previous nutritional analysis of food blogs, which found that the average protein content of vegetarian recipes across six food blogs provided only 27% of the recommended daily amount of protein, compared to 57% across all recipe types combined [64]. However, vegetarian food blogs run by registered dietitians have been found to be significantly lower in energy and sodium than non-RDs [21]. This suggests that the difference lies in the background knowledge and nutritional education of the recipe author, again highlighting the impact of relevant qualifications.

The medical practitioner in the current study only scored 46%, possibly reflecting the lack of nutrition within UK medical degrees, something AfN and Royal Colleges have been working to improve. When surveyed, only 21% of UK medical students were confident in their knowledge of UK dietary guidelines [67]. The British public consider doctors to be the most trusted profession [37]; however, inadequate advice may lead to loss of trust, perhaps explaining why the public seek nutritional advice elsewhere. Doctorates and other qualifications may also be purchased online, and thus therefore might not always reflect credibility. Moreover, no qualified influencer provided a clearly accessible disclaimer, reflecting an earlier study where less than half of bloggers presented a disclaimer, not differing significantly between registered dietitians (RDs) or non-qualified bloggers [20]. The study was limited, however, categorising qualified nutritionists as unqualified. The lack of qualified influencers in this cohort reflect a survey of 679 food bloggers, where only 22% had a professional history in food, and not necessarily as nutritionists or dietitians [68].

Sharing personal stories promotes trust [69], making SMI appear more vulnerable and relatable. This technique is used by SMI to build brands and increase follower engagement [10]. Lepkowska-White and Kortright describe this in their analysis of food bloggers, finding it to be part of the business strategy used to build credibility and relatability [70]. As their study only included female bloggers, it is unclear whether males follow the same approach; however, exploration of this may uncover different strategies. In the current study, four female SMI detailed personal stories of using diet to overcome illness, followed by a desire to share this with the public. People may read such stories and try to ‘cure’ themselves, without consulting a qualified professional. It is imperative that advocated diets are therefore transparent as to their effects and are scientifically backed.

### 4.2. Strengths and Limitations

The main strength of this study is novel insight into the credibility of WM SMI blogs which may inform the methodological approach for future studies. To the best of our knowledge, no previous study has applied content analysis to this topic. The results suggest that it is paramount that the information offered by SMI online is examined, as failing to consider this area in government policy may lead to loopholes that can be easily exploited, resulting in widespread unregulated misinformation. This may, in turn, have implications for public health practitioners, organisations and mass education campaigns.

Influencers, legally, must declare paid-for content prominently in online posts; however, violations still occur [71]. Governments might consider introducing a credibility award to display on blogs, something that is currently being proposed in a petition to Government by Registered Nutritionists in the UK, supported by the British Dietetic Association [72]. Subsequent research should focus on developing the methodological approach for assessing nutrition and WM information supplied online by SMI, to gather more data required to inform policy and practice.

This study also sought to test the ability of a newly developed checklist to assess the credibility of WM blogs, as none currently exist to our knowledge. Previously, nutritional quality has been the sole focus but other aspects, such as qualifications, referencing, opinion versus fact and disclaimers were investigated. The checklist offered initial patterning of WM information provided online by SMI, providing novel insight into an important area of research. Binary questions gave a clear and simple way of determining aspects of credibility, but non-binary answers were harder to determine. For example, an adherence cut-off was required for certain indicators, set at a potentially generous 70%. The previous literature has no adherence cut-offs, therefore future development should seek to determine if this cut off is most appropriate or if other thresholds or methods are more suitable.

Due to the binary nature of questions, some SMI were given a ‘0’ when the blog did not contain, e.g., information-based posts. Although this ensured that scores for SMI were only based on the elements they displayed on their blogs, it could potentially introduce inaccuracies, owing to the fact that elements such as having no advertising at all on a blog do not necessarily have any implication on credibility. This is an issue to be resolved when the methodology is developed further. 

The online environment changes rapidly and this study provides a ‘snapshot’ in time. The PHE calorie guidelines focus on meals as a unit with little advice about snacks; however, adults might consume additional foods. Resource availability and timescale meant that the sample and number of meal recipes analysed was relatively small, but it was judged enough to reflect the blog tone and content. Additionally, previously published research has analysed fewer blogs (5) than the current study over a three month timeframe [18]. Research following this study should consider a larger sample over a longer time period to examine trends. For example, as has been observed in TV food advertising [73], there may be similar shifts due to patterns associated with seasons, e.g., Christmas, Easter, BBQ season. 

Additionally, having only one coder is a limitation. In future, two coders should be used when analysing larger volumes of data (e.g., a greater number of blog posts), with consistency checks.

## 5. Conclusions

WM-related blogs published by UK SMI may not be credible resources for WM. These findings highlight the need for more extensive evidence-based data on this topic to inform any regulation of online WM information, and development of a robust means of assessing the credibility of SMI blogs. Policy intervention may be required to suppress the spread of online misinformation that may undermine efforts to achieve and maintain a healthy diet and body weight, and support consumers to make informed healthy dietary choices.

## Figures and Tables

**Table 1:** Credibility checklist of quality indicators.

Theme	Comments
**Transparency**	
Is the identity of the author always clear?	Either explicitly stated or implied through use of personal pronouns.
Is there a professional statement present?	A note of their background and experience in the field.
Does the influencer respect and protect the privacy of blog readers?	Is there a clearly accessible privacy statement? Normally situated in the footer and should be accessible from every page. Must be specific to the blog and protection of readers/subscribers.
Are appropriate disclaimers regarding the use of the blog stated clearly?	Are these clearly accessible from the blog? Normally situated in the footer and should be accessible from every page.
**Use of Other Resources**	
Are references cited on the blog?	Based on the first 20 information-based posts. 70% to pass.
Are the references always evidence-based?	Based on the first 20 information-based posts. 70% to pass.
**Trustworthiness and Adherence to Nutritional Criteria**	
Is the author well qualified to provide weight management information?	‘Well qualified’ for this purpose means that they hold a relevant nutrition or dietetics degree or are a medical doctor.
Has the influencer been awarded any quality credentials/badges from independent organisations?	From official nutrition or dietetics organisations, not including holistic or nutritional therapy credentials.
Do the recipes in the blog adhere to UK nutritional criteria (PHE kcal)? Do the recipes in the blog adhere to UK nutritional criteria (FSA traffic lights)?	70% to pass, to ensure ‘adherence’. 70% to pass, to ensure ‘adherence’
**Bias**	
Does the blog clearly differentiate between advertisement and content?	Sponsored posts, including paid promotions/partnerships, Google ads, advertising of products and external services, affiliate links.
If the blog includes external advertising, is it disclosed?	Sponsored posts, including paid promotions/partnerships, Google ads, advertising of products and external services, affiliate links.
Does the influencer always make clear distinction between fact and opinion on their blog?	Linked to the citation of references.

**Table 2:** Influencers who advocated specific diets.

Influencer	SMI-3	SMI-4	SMI-9	SMI-8
Diet	Gluten-Free	Gluten-free	Gluten-free	Gluten-free
	Wheat-Free	Grain-free	Whole foods	Low grain
	Dairy-Free	No hydrogenated oils	No processed foods	Dairy-free
	Refined Sugar Free	Refined sugar free	Plant-based/vegan	Refined sugar-free
	Plant-based			Paleo

**Table 3:** Calorie information for breakfast, lunch and dinner recipes, with means and as a percentage of PHE target.

Influencer	Breakfast		Lunch		Dinner	
	Mean kcal	% PHE Target	Mean kcal	% PHE Target	Mean kcal	% PHE Target
	(Range)		(Range)		(Range)	
SMI-1	420	105	479	80	650	108
	(195–662)		(360–569)		(512–845)	
SMI-2	695	174	840	140	517	86
	(615–851)		(690–982)		(419–647)	
SMI-3	517	129	642	107	544	91
	(287–861)		(516–805)		(314–992)	
SMI-4	495	124	596	99	480	80
	(250–651)		(524–686)		(356–764)	
SMI-5	623	156	684	114	843	140
	(280–1062)		(424–1024)		(486–1592)	
SMI-6	457	114	426	71	454	76
	(293–594)		(269–640)		(234–641)	
SMI-7	460	115	479	80	471	79
	(387–593)		(353–623)		(312–641)	
SMI-8	236	59	475	79	429	71
	(130–395)		(246–915)		(263–672)	
SMI-9	481	120	551	92	623	104
	(232–630)		(214–987)		(374–883)	

**Table 4:** Percentage of nutritional criteria adherence (% of meals that fell within PHE kcal limits, % of meals that fell within traffic light criteria) for each influencer.

Influencer	PHE Kcal Limit (%)	Traffic Lights (%)
SMI-7	90	78
SMI-8	80	73
SMI-6	80	63
SMI-1	70	65
SMI-3	70	60
SMI-4	60	58
SMI-9	50	70
SMI-5	50	43
SMI-2	40	53

**Table 5:** Mean macronutrient composition of influencer recipes.

Influencer	Mean Macronutrient Composition (% and Range)		
	Carbohydrate	Protein	Fat
SMI-1	33 (7.7–56.5)	26 (12.2–46.7)	40 (23.9–56.5)
SMI-2	35 (11.4–47.8)	35 (21.1–52.2)	30 (14.4–49.4)
SMI-3	48 (14.7–69.5)	13 (6.3–19.3)	39 (12.9–67.5)
SMI-4	15 (2.8–27.8)	25 (4.8–26.5)	60 (33.9–81.5)
SMI-5	33 (10.5–77.9)	19 (4.8–32)	48 (11–70.2)
SMI-6	43 (9.7–75.3)	19 (7.8–44)	37 (11.3–61.5)
SMI-7	24 (3.5–57.2)	30 (19–41.1)	45 (9–70.2)
SMI-8	18 (3.8–36.5)	29 (13.9–40.9)	53 (26.5–73.1)
SMI-9	47 (21.4–72.2)	12 (7.9–17.6)	41 (11–64.2)

## Supplementary Materials

The following are available online at https://www.mdpi.com/1660-4601/17/23/9022/s1, Table S1: Full breakdown of themes and quality indicators used in the analysis. Full results are displayed alongside percentage pass and fail rates for each influencer.
