# Supplementary material for: Analysing Credibility of UK Social Media Influencers’ Weight-Management Blogs: A Pilot Study
Source: Int J Environ Res Public Health. 2020 Dec 3;17(23):9022. doi: 10.3390/ijerph17239022 (PMC7731114; doi:10.3390/ijerph17239022)
Supplement: Supplementary file 1 [file ijerph-17-09022-s001.pdf]

Table S1. Full breakdown of themes and quality indicators used in the analysis. Full results are displayed alongside percentage pass and fail rates for each influencer

Theme	Influencer								
Transparency	SMI-1	SMI-2	SMI-3	SMI-4	SMI-5	SMI-6	SMI-7	SMI-8	SMI-9
Is the identity of the author always clear?	Yes	Yes	Yes	Yes	Yes	Yes	Yes	Yes	Yes
Is there a professional statement present?	No	Yes	No	No	Yes	Yes	Yes	Yes	No
Does the influencer respect and protect the privacy of blog readers?	Yes	Yes	No	No	Yes	No	No	Yes	No
Are appropriate disclaimers regarding the use of the blog stated clearly?	Yes	Yes	No	Yes	No	No	No	Yes	No
Use of other resources									
Are references cited on the blog?	No	No	Yes	0	No	No	Yes	No	0
Are the references evidence-based?	No	No	Yes	0	No	No	Yes	No	0
Trustworthiness and adherence to nutritional criteria									
Is the author qualified to provide weight management information?	No	No	No	No	No	Yes	Yes	No	No
Has the influencer been awarded any quality credentials/badges from independent organisations?	No	No	No	No	No	No	Yes	No	No
Do the recipes in the blog adhere to UK nutritional criteria (PHE)?	Yes	No	Yes	No	No	Yes	Yes	Yes	No
Do the recipes in the blog adhere to UK nutritional criteria (FSA)?	No	No	No	No	No	No	Yes	Yes	Yes
Bias									
Does the blog clearly differentiate between advertisement and content?	Yes	Yes	Yes	Yes	No	Yes	Yes	Yes	Yes
If the blog includes external advertising, is it disclosed?	Yes	No	Yes	Yes	No	Yes	Yes	Yes	Yes
Does the influencer always make clear distinction between fact and opinion on their blog?	No	No	No	0	No	No	Yes	No	0
Yes (%)	46%	38%	46%	40%	23%	46%	85%	61%	40%
